# Effects of Partial Blackwater Substitution on Soil Potential NI-Trogen Leaching in a Summer Maize Field on the North China Plain

**DOI:** 10.3390/life12010053

**Published:** 2021-12-31

**Authors:** Tao Zhang, Hao Peng, Bo Yang, Haoyu Cao, Bo Liu, Xiangqun Zheng

**Affiliations:** 1 Aerospace Environmental Engineering Co., Ltd., Tianjin 300301, China; 2516209097@tju.edu.cn; 2 Agro-Environmental Protection Institute, Ministry of Agriculture and Rural Affairs, Tianjin 300191, China; penghao@caas.cn (H.P.); 82101205238@caas.cn (H.C.); 82101185126@caas.cn (B.L.); zhengxiangqun@caas.cn (X.Z.); 3 School of Environmental Science and Engineering, Tianjin University, Tianjin 300072, China

**Keywords:** nitrogen leaching, blackwater, wastewater reuse, maize fertilization, N surplus

## Abstract

In China, promoting harmless blackwater treatment and resource utilization in rural areas is a priority of the “toilet revolution”. Exploring the effects of blackwater application in arid areas on soil nitrogen losses can provide a basis for more effective water and fertilizer management. This study analyzed nitrogen leaching and maize yield under blackwater application in the summer maize season of 2020. A total of 5 treatments were used: no fertilizer, single chemical fertilizer application (CF), single blackwater application (HH), and combined chemical fertilizer and blackwater application ratios of 1:1 (CH1) and 2:1 (CH2). The total nitrogen leached from the fertilization treatments was 53.14–60.95 kg·ha^−1^ and the leached nitrate nitrogen was 34.10–40.62 kg·ha^−1^. Nitrate nitrogen accounted for 50–62% of the total leached nitrogen. Compared with blackwater treatments, nitrate nitrogen moved into deeper soil layers (80–100 cm depth) during the CF treatment. Compared with CF, HH significantly reduced the maize yield by 24.39%. The nitrogen surplus of HH was higher than that of other fertilizer treatments. Considering nitrogen leaching, maize yield, and economic benefits, the CH2 treatment presented the optimal results. These findings address knowledge gaps and assist in guiding policy-makers to effectively promote China’s “toilet revolution”.

## 1. Introduction

Farmers worldwide have long used human excrement as a quick-acting fertilizer, owing to its high nitrogen content [1]; this is a traditional practice which has been followed over generations [2]. In China, the “toilet + septic tank + blackwater utilization” model is widely used to prevent pollution and promote recycling of human excrement [3]. In this model, after toilet sewage enters the septic tank, the decomposed manure liquid (i.e., blackwater) is used as a fertilizer for crops [4]. A previous study showed that blackwater use can improve soil structure and porosity while increasing soil organic carbon, and that reusing blackwater as a fertilizer for agriculture can help address soil productivity issues [5]. However, toilet flushing water dilutes the nutrient content of blackwater; therefore, considerably more blackwater is required to ensure normal crop growth, which increases the risk of nutrient loss.

After nitrogen fertilizers are applied to farmland soils, their fate can be roughly divided into three parts: some nitrogen is transformed into effective nutrients and is absorbed and utilized by the crops [6]; some is fixed in the crystal lattice of soil minerals, and thus remains in the soil [7]; the remainder is lost through leaching, nitrification, and denitrification [8,9]. In China, the overall utilization efficiency of nitrogen fertilizers in agriculture is only 30–40% [10], and the data of the first national pollution census showed that total nitrogen loss from agricultural sources accounted for 57.2% of total emissions in China [11]; therefore, significant economic losses are caused by this inefficiency.

Gradual nitrogen leaching below the root zone (i.e., nitrogen mineralization is not synchronized with nitrogen absorption by plants) is an important N-loss pathway which easily occurs in the presence of rainfall and irrigation events [12]. Soil is mostly composed of negatively charged colloids; therefore, it easily adsorbs a large amount NH_4_^+^-N, whereas the adsorption of NO_3_^−^-N is weak [13]. Therefore, the nitrogen element in the soil easily moves vertically downward with water in the form of NO_3_^−^-N, which characterizes the nitrogen leaching and represents approximately 60% of total dissolved nitrogen loss [14]. Nutrients that leach out of the active layer of plant roots are not easily absorbed by plants, which greatly reduces the nutrient use efficiency of the soil. If the leached nitrogen flows into groundwater, it can lead to exceedingly high levels of nitrate, which can endanger human health.

It has been previously reported that nitrification is stronger in alkaline than acid and neutral soils [15]; therefore, higher concentrations of NO_3_^−^-N increase the risk of nitrogen migration in soil. Moreover, nitrogen movement in soil is not only controlled by the soil environment and hydrological processes, but also by crops and management measures [16,17,18]. The North China Plain is China’s main dryland food production area, with alkaline soil, low water-holding capacity, low organic-matter content, and weak fertilizer-retention capacity [19]. Large amounts of fertilizer and irrigation water are needed to achieve a relatively high yield in these areas [20,21]. However, the use of blackwater as fertilizer may lead to a lower nitrogen utilization rate and higher nitrogen leaching due to the high moisture content [4]. In Beijing-Tianjin-Hebei and other intensive cultivation areas, >40% of the groundwater has a nitrogen content higher than the country’s standards for drinking water (the Standards for Drinking Water Quality of China for NO_3_^−^-N (GB5749-2006) is 20 mg·L^−1^) [22]. However, to the best of our knowledge, there has been no research on nitrogen leaching from blackwater that is returned to fields in these areas.

In this study, we aimed to identify the potential environmental risk of blackwater for agriculture utilization and offer an available strategy to recover the energy and nutrients provided by blackwater. The main objectives of this experiment were to: (i) evaluate the effects of blackwater application levels on nutrient loss and crop yield in the alkaline soils of North China and (ii) explore the threshold of blackwater input under natural rainfall conditions in North China. The findings provide a reference to guide the “toilet revolution”, reduce nitrogen loss, and decrease non-point source pollution from farmland.

## 2. Materials and Methods

### 2.1. Research Area Overview

The study area was located in a maize field (39°33′ N, 117°82′ E) in Dongjiituo Township, Ninghe County, Tianjin. The area has a continental monsoon climate and is in a warm temperate climatic and semi-arid, semi-humid wind zone, with relatively high summer temperatures and concentrated precipitation, as well as relatively cold and dry winters. The annual mean temperature is 11.2 °C; the minimum and maximum temperatures occur in January and July, respectively; the annual frost-free period is 240 d. Annual mean precipitation is approximately 642 mm, which mainly occurs between June and August, accounting for 70% of the annual precipitation. The cultivated soil in the study site was fluvo-aquic. The basic physical and chemical properties of the soil were as follows: pH = 8.38; organic matter = 9.70 g·kg^−1^; total nitrogen (TN) = 1.19 g·kg^−1^; total phosphorus (TP) = 0.64 g·kg^−1^; alkali hydrolyzable nitrogen = 81.30 mg·kg^−1^; available phosphorus = 23.05 mg·kg^−1^; cation exchange capacity = 16.3 cmol·L^−1^.

### 2.2. Experimental Setup

A randomized block design was used in the experiments. The chemical fertilizers used were urea (N = 46%), superphosphate (P_2_O_5_ = 16%), and potassium oxide (K_2_O = 60%). The blackwater utilized was the effluent from a 3-grid septic tank (N: 5.1 g·kg^−1^; P: 3.1 g·kg^−1^; K: 3.7 g·kg^−1^). A total of 5 fertilizer treatments were explored: no fertilizer (CK); single application of chemical fertilizer (CF); single application of blackwater (HH); combined application of chemical fertilizer and blackwater at ratios of 1:1 (CH1) and 2:1 (CH2). The experiments for each treatment were repeated four times. The area of a single test plot was approximately 24 m^2^ (4 m × 6 m), and a completely randomized block arrangement design was used.

The amount of fertilizer applied to the crops grown in the test site was determined based on the local fertilizing habits. In the summer maize season, the nitrogen application rate was 200 kg·ha^−1^, the phosphorus application rate was 150 kg·P·ha^−1^, and the potassium application rate was 150 kg·K·ha^−1^. The chemical fertilizers P_2_O_5_ and K_2_O were used to remediate insufficient blackwater phosphorus and potassium contents, and were applied as a base fertilizer on a single occasion (Table 1). Blackwater and chemical nitrogen fertilizers were both applied to the surface at a ratio of 4:6.

### 2.3. Sample Collection

#### 2.3.1. Leachate Samples

**Figure 1:**
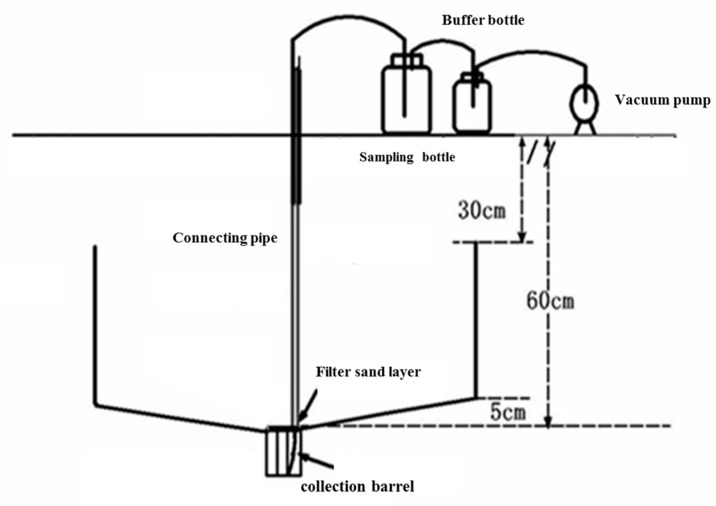
Leaching sample collection device and sampling scene.

In this experiment, infiltration tanks were used for in-situ monitoring of soil leachate (Figure 1). The leachate collection device was buried in each treatment plot in October 2019, and the leaching tube was planted and domesticated after a crop of winter wheat. The upper part of the collection device was composed of a sampling bottle, a buffer bottle, a vacuum pump, and a connecting pipe, and the underground part was composed of a filter sand layer, a liquid-collecting film, and a leachate collection barrel. The filter sand layer was composed of quartz sand with particle size of 2–3 mm, which was repeatedly cleaned with diluted acid and water. The liquid-collecting film included 2 pieces of polyethylene film with a 0.1-mm thickness. The leachate collection barrel was a cylindrical water barrel composed of a polyethylene material, with a volume of approximately 69 L (50 cm in diameter, 35 cm in height), and was buried at a depth of 80 cm.

We used a vacuum pump to generate negative pressure and extract all of the leachate for analysis. After evenly mixing the leachate samples, 500 mL of the sample was placed in a washed and dried polyethylene bottle and stored at 4 °C. A continuous flow injection analyzer (AA3 HR Auto Analyzer, SEAL Analytical, Germany) was used to determine the TN, NH_4_^+^, and NO_3_^−^ contents in the eluent within 24 h of the collection. The sampling time was determined according to rainfall events, and the leachate samples of all sampling points were collected within 1 d. A total of 7 sampling campaigns were conducted throughout the experiment, on the day of 15 July, 30 July, 4 August, 15 August, 24 August, 19 September, and 19 October in 2020. Temperature and rainfall information were obtained from a small weather station.

#### 2.3.2. Plant Sampling

The test crop was a summer maize variety, Jingdan 58, the main local variety. The crop was sown on 27 June 2020, with row spacing of 60 cm and plant spacing of 25 cm. The crop was harvested on 15 October 2020 after a 110-d growth period. The maize yield of each plot was determined. In addition, 3 representative plants were randomly selected from each plot. The dried samples were ground into powder and passed through a 100-mesh sieve. After digestion with concentrated H_2_SO_4_-H_2_O_2_, the TN content was determined by the semi-micro Kjeldahl method according to the maize yield. Subsequently, the nutrient absorption of the maize was calculated based on its nutrient content.

### 2.4. Data Analysis

The cumulative nitrogen (TN, NH_4_^+^, and NO_3_^−^) leaching amount was calculated according to Equation (1) [23].

(1) $$N_{L}=\sum_{i=1}^{n}\frac{\left(C_{i}\times V_{i}\times 10^{-3}\right)}{1\times 10^{-2}\times 10^{-4}}$$

where *N_L_* represents the *N* loss loadings via surface runoff or leaching (kg·ha^−1^), *Ci* represents the *N* concentration of the water sample of each leaching sampling (mg·L^−1^), *Vi* represents the water volume of each leaching sampling (*L*), and 1 × 10^−2^ is the monitoring area (m^2^).

The nitrogen surplus was estimated from total harvested nitrogen and all of the nitrogen inputs based on nitrogen balance in the summer maize cropping system [24]. The nitrogen surplus was calculated according to Equation (2):

(2) N surplus=input N−output N

where the main external nitrogen inputs in our experiment were nitrogen brought by chemical fertilizer and blackwater. Other inputs, such as nitrogen from atmospheric deposition and irrigation water, were ignored. Nitrogen output included the nitrogen harvested in aboveground biomass (shoots and grains).

All of the statistical analyses were carried out in JMP version 9.0 (SAS Institute Inc., Cary, NC, USA, 2010). All of the data were checked for homogeneity of variances (Levene’s test) and normality (Shapiro–Wilk test), and were normally distributed and had homogeneous variances. Differences among treatments in crop yields, nitrogen uptake, nitrogen surplus, nitrate distribute and cumulate nitrogen leaching were further examined with Student’s multiple range tests. The effects of soil profiles and fertilization treatments on nitrate content distribution were examined by two-way analysis of variance (ANOVA). Origin 2019 was used to draw the soil nitrate distribution in the soil profile.

## 3. Results

### 3.1. Rainfall Characteristics

**Figure 2:**
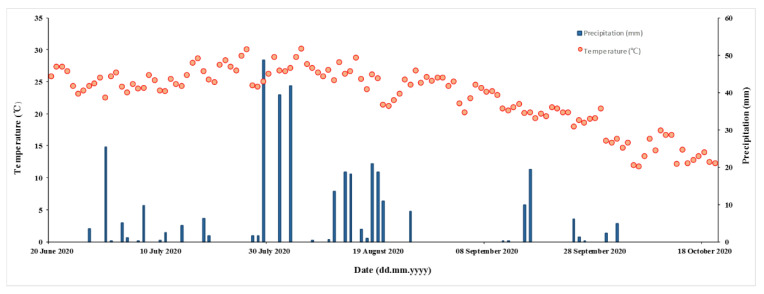
Daily precipitation (mm) and temperature (°C) from 20 June 2020 to 20 October 2020.

The maize growing season is the wet season in the basin, with a total rainfall of 350.4 mm; this accounts for 72.3% of the annual rainfall. There were 5 rainfall events with precipitation of >20 mm (Figure 2). The largest rainfall event during the study period occurred on 29 July 2020 and reached 48.7 mm, accounting for 10.1% of the annual rainfall.

### 3.2. Maize Production and Nitrogen Surplus

**Figure 3:**
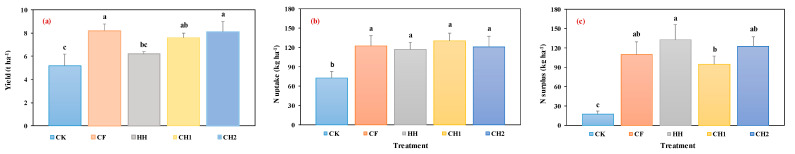
(**a**) Grain yield, (**b**) nitrogen uptake, and (**c**) nitrogen surplus of maize under different treatments. CK: no fertilizer, CF: chemical fertilizer, HH: blackwater, CH1: combined application of chemical fertilizer and blackwater at ratio of 1:1, CH2: combined application of chemical fertilizer and blackwater at ratio of 2:1. Bars indicate the standard error of the mean (+SE) for three replicates of each treatment. Letters above columns indicate significant differences according to the Tukey’s multiple range test (*p* < 0.05) among all treatments.

The results of the experiments showed that the yield of maize kernels under different treatments was 5.2–8.2 t·ha^−1^, with an average yield of 7.1 t·ha^−1^ (Figure 3a). Compared with the CF treatment, the combined treatments presented no significant effects on the maize yield, whereas the HH treatment significantly reduced the maize yield by 24.4% (*p* < 0.05). For the treatments using blackwater, a higher proportion of chemical fertilizer led to a higher maize yield; therefore, the maize yield under HF2 was significantly higher than that under HH, with an increase of 23.5% (*p* < 0.05), although there was no significant difference between the 2 combined treatments.

The nitrogen uptake of maize under different treatments ranged from 72.4–130.0 kg·ha^−1^ (Figure 3b). Compared with the CK treatment, the 4 fertilization treatments significantly increased the nitrogen uptake in the aboveground part of maize; however, there was no statistical significance among the 4 fertilizer treatments (*p* > 0.05). An analysis of nitrogen surplus showed that the nitrogen surplus of each fertilization treatment was significantly higher than that of the CK treatment (Figure 3c). Among the fertilization treatments, the HH treatment had the largest nitrogen surplus, reaching 132.4 kg·ha^−1^, which was significantly higher than that of the CH1 treatment (51.2%; *p* < 0.05).

### 3.3. Nitrate Nitrogen Migration in Soil Profile

**Figure 4:**
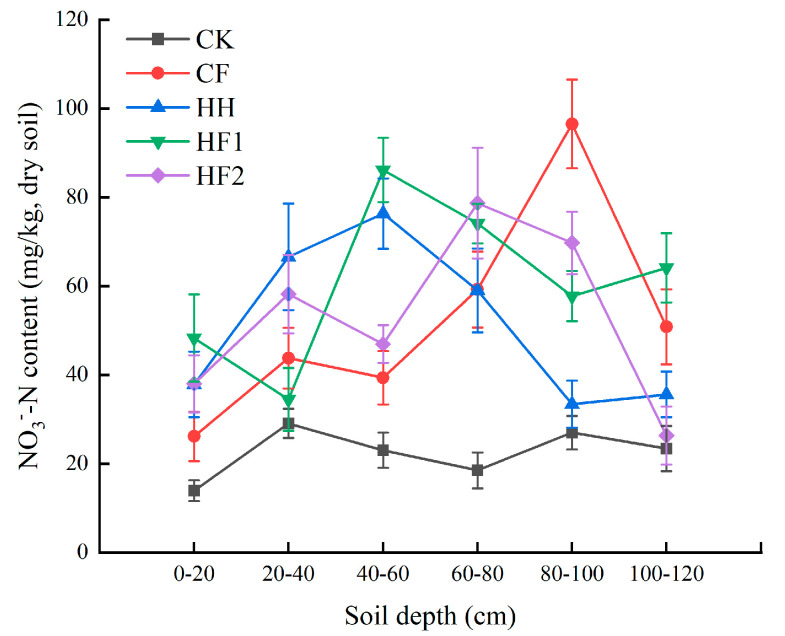
Nitrate nitrogen distribution in different soil profiles. Bars indicate the standard error of the mean (+SE) for three replicates of each treatment. CK: no fertilizer, CF: chemical fertilizer, HH: blackwater, CH1: combined application of chemical fertilizer and blackwater at a ratio of 1:1, CH2: combined application of chemical fertilizer and blackwater at a ratio of 2:1.

Figure 4 shows the soil nitrate nitrogen profile at a depth of 0–120 cm under different fertilization strategies. The 3 treatments with blackwater application (HH, HF1, and HF2) reached the highest nitrate nitrogen content at a 40–80 cm depth. The highest nitrate nitrogen content of CF was observed at a depth of 80–100 cm, which indicates that the nitrogen from chemical fertilizers leached more easily downward. This result was supported by the one-way ANOVA. 

The nitrate nitrogen content in the soil profile and its spatial distribution characteristics are important indicators to characterize the leaching risk (Table 2). At a depth of 80–100 cm, the nitrate nitrogen content of the CF treatment was significantly higher than those of the blackwater application treatments, which were 63.4% (HH), 40.1% (HF1), and 27.7% (HF2) (*p* < 0.05). However, there was no statistical difference between the accumulation of nitrate nitrogen in the 4 fertilized soils at a depth of 60–80 cm (*p* > 0.05). The two-factor ANOVA showed that the fertilization strategy and soil depth significantly affected nitrate nitrogen leaching (Table 2). 

### 3.4. Nitrogen Leaching

Table 3 shows the different forms of nitrogen in the leachate of each treatment. The total nitrogen leached from the different treatments was 34.91–60.95 kg·ha^−1^. Compared with CK, all of the fertilization treatments significantly increased the amount of leached nitrate nitrogen and TN, with increases of 57.80–87.97% and 52.22–74.59%, respectively (*p* < 0.05). However, there was no significant difference among the four fertilization treatments. Compared with the other treatments, the amount of leached ammonium nitrogen was significantly higher in the HH treatment. The amount of leached nitrate nitrogen accounted for 17.05–20.31% of the total leached nitrogen, and the leaching rate of ammonium nitrogen was relatively small, accounting for 0.38–1.41% of the total nitrogen leached from all the different treatments.

## 4. Discussion

### 4.1. Effect of Fertilization Strategies on Nitrogen Leaching

Leaching is an important mechanism of nitrogen fertilizer loss, and the form of fertilizer used is an important farmland management measure affecting nitrogen leaching. In this study, compared with CF, nitrogen leaching decreased by 7.6–12.8% in the 3 treatments based on blackwater, although there were no statistically significant differences. Previous studies have shown that nitrogen leaching may vary with fertilizer type [25]. Under the same nitrogen application levels, the combined application of organic and inorganic fertilizers can significantly reduce nitrogen leaching (the content of organic matter in blackwater is higher than that in chemical fertilizer) compared to single application of chemical fertilizers [26]. This is due to the inherent ability of organic matter to improve the soil quality, increase the soil water retention capacity, and promote crop nitrogen uptake [27,28]. Studies have also shown that a high C/N ratio helps to promote the conversion of mineral nitrogen to organic forms (i.e., nitrogen immobilization.) [29,30], thereby reducing nitrogen leaching and runoff.

Interestingly, although no fertilizers were used in the CK treatment, nitrogen leaching of 34.91 kg·ha^−1^ occurred, which we presumed was due to: (i) nitrogen fertilizer remaining from previous crops, since residual nitrate can move continuously downwards and be lost even if it is not leached during the season of application [11]; (ii) nitrogen deposition, for example, a 3-year study investigated atmospheric deposition of different nitrogen species at 10 sites in Northern China and the results indicated that nitrogen deposition levels in Northern China were high, with an average of 59.8 kg·N·ha^−1^·yr^−1^ [31].

### 4.2. Effect of Fertilizer with Blackwater on Nitrate-Nitrogen Migration

In our study, the NO_3_^−^ leaching of the 4 fertilization treatments accounted for approximately 17.05–20.31% of the fertilizer input, which was slightly higher than some other studies, such as a meta-analysis conducted by Zhou & Butterbach-Bahl [32], who collected 32 published studies reporting NO_3_^−^ leaching losses in maize and determined that 15% of applied fertilizer nitrogen to maize systems worldwide are leached in the form of NO_3_^−^. However, our obtained results are within the value estimated by Cui et al. [33], which conducted a meta-analysis of 17 published studies from 19 study sites, including 94 observations from maize system in China, and found that with typical farming practices, an average of 20.8% of the applied nitrogen was either leached or lost as runoff from the maize systems. The difference between the results may be attributed to soil physico-chemical properties such as texture, pH [34], soil organic carbon [35], crop type [25], or annual precipitation. It is worth mentioning that the residual NO_3_^−^ in the soil profile showed that the NO_3_^−^ leaching depth was deeper in the CF treatment, with a peak value at the 80–100-cm depth. The root system of the maize plats was mainly concentrated in the soil layer above 90 cm, which indicated that CF was more likely to cause NO_3_^−^ leaching to groundwater.

The total nitrate accumulations in the 0–4 m soil layer of maize fields was as high as 749 ± 75 kg·N·ha^−1^ in China [36]. However, the average accumulation of nitrate in the 0–120 cm soil layer was 51.5–60.8 kg·nitrogen·ha^−1^ in this study, considerably lower than the national average value. There are 3 possible reasons that may explain this finding: (1) the nitrogen application rate in our experiment (200 kg·nitrogen·ha^−1^) was lower than the typical rates (263 kg·nitrogen·ha^−1^) for wheat in the North China Plain [37]; (2) we conducted the experiment in the rainy season of the North China Plain, which facilitated the rapid transport of nitrate deeper into the underground water [38]; (3) the soils have high permeability and low cation exchange capacity [20].

### 4.3. Effects of Blackwater Application on Soil Nitrogen Surplus and Maize Yield

Nitrogen surplus is an effective indicator for measuring nitrogen input productivity, environmental impact, and soil fertility changes [39]. Maintaining the nitrogen balance of the soil-crop system can achieve higher target yields without consuming soil nitrogen. In our study, the highest nitrogen surplus was obtained in the HH treatment, which may have been due to this treatment yielding the lowest maize yield of the four fertilizer treatments. The lowest nitrogen surplus in CH1 treatment may have been related to its high nitrogen uptake. 

Chemical fertilizers (especially nitrogen fertilizers) are applied at high rates for food production in China, which leads to decreases in crop yield and quality, and an increase in fertilization costs [40]. This study showed that the application of pure blackwater significantly reduced maize yield compared to the application of conventional fertilizer, which may have been attributed to the higher content of base ions in blackwater [41]. Studies have shown that maize is susceptible to soil salinity, which significantly decreases seed germination, causes harmful effects in growth, and leads to low yield [42]. However, in this study, the yields under the combined treatments were not significantly different from that under CF, which indicated that an appropriate amount of blackwater can maintain the maize yield. Nitrogen fertilizer is a costly component of crop production [43], and at present, the average price of chemical fertilizers in China is 3 yuan/kg. Therefore, according to our results, the use of a combined fertilization treatment can lead to savings of approximately 190–300 yuan/ha, which would reduce the economic burden of fertilization to farmers.

### 4.4. Feasibility and Prospect of Returning Blackwater to the Field

A large-scale survey revealed that the proportion of pathogenic bacteria in the effluent from septic tanks is very low [44], and ensured environmental health and agricultural application safety. While approximately 86% of the households stated that they would prefer their excreta to be used in agriculture as fertilizer [45], there is no instructional document to teach farmers how to use blackwater to fertilize, and the usual practice of farmers is to return all the collected blackwater to the field. However, according to our study, excessive blackwater may lead to reduced crop production, which has a huge impact on farmers. A balance is needed between increasing farmers’ income and decreasing environmental impact, and the fertilizer strategy involving the CH2 treatment appeared to meet both requirements.

In addition, our previous study shows that the application of a reasonable proportion of blackwater and chemical fertilizers did not significantly increase reactive nitrogen emissions [46]. However, application of blackwater-based fertilizers in agriculture will alleviate the environmental impacts of phosphorus mining and synthetic ammonia production [47]. In summary, exploration of the means by which to recycle blackwater or using excreta-derived fertilizers in agriculture is urgently for decision makers.

## 5. Conclusions

In summary, this study showed that compared to chemical fertilizer, blackwater application could prevent nitrate nitrogen from moving to deeper soils (below 80 cm), and that there was no statistical difference in soil nitrogen surplus and crop nitrogen uptake. Furthermore, the blackwater fertilizer strategy decreased the nitrate nitrogen and total nitrogen leaching by 7.4–16.1% and 7.6–12.8%, respectively. However, the application of blackwater at 200 kg·nitrogen·ha^−1^ reduced the maize yield by approximately 24.4% compared to application of chemical fertilizer, which may have been due to the high salt content of blackwater. The combined application of blackwater and chemical fertilizers maintained the maize yield without increasing the risk of nitrogen leaching, especially when the ratio of chemical fertilizer to blackwater was 2:1 (i.e., chemical fertilizer provided 133 kg·nitrogen·ha^−1^, blackwater provided 67 kg·nitrogen·ha^−1^). Our study shows that a potential reduction in nitrogen leaching and obtainable high maize yield can be achieved by the appropriate blackwater substitution of chemical fertilizers. We suggest that promoting the return of blackwater to fields not only involves allowing farmers to utilize it as fertilizer, but also includes introducing, demonstrating, and teaching them how it can be optimally carried out.

## Figures and Tables

**Table 1:** Fertilizer application rates of experimental treatments at different growth stages of maize (kg·ha^−1^).

Treatments	Applied Fertilizers Rate (kg·ha^−1^)			Fertilizer Form	Application Date
	N	P	K		
CK	-	-	-	-	-
	-	-	-	-	
CF	80	150	150	urea + superphosphate + potassium oxide	21 June 2020
	120	-	-	urea	
HH	80	29 + 48	5 + 58	blackwater + superphosphate + potassium oxide	
	120	73	87	blackwater	
CH1	40 + 40	40 + 24	77 + 29	urea + blackwater + superphosphate + potassium oxide	3 August 2020
	60 + 60	36	44	urea + blackwater	
CH2	53 + 27	110 + 16	101 + 20	urea + blackwater + superphosphate + potassium oxide	
	80 + 40	24	29	urea + blackwater	

CK: no fertilizer, CF: chemical fertilizer, HH: blackwater, CH1: combined application of chemical fertilizer and blackwater at 1:1, CH2: combined application of chemical fertilizer and blackwater at 2:1.

**Table 2:** Nitrate nitrogen content in different soil profiles. CK: no fertilizer, CF: chemical fertilizer, HH: blackwater, CH1: combined application of chemical fertilizer and blackwater at a ratio of 1:1, CH2: combined application of chemical fertilizer and blackwater at a ratio of 2:1.

Soil Profile (cm)	CK	CF	HH	CH1	CH2	Two-Way ANOVA
0–20	13.9 ± 2.4bC	26.1 ± 5.6cBC	37.8 ± 7.4bcAB	48.3 ± 9.9cdA	37.9 ± 6.4bcAB	Treatment (T)
20–40	29.1 ± 3.3aC	43.8 ± 6.8bcBC	66.6 ± 12.0aA	34.5 ± 7.1dC	58.2 ± 8.8abAB	*p* < 0.001
40–60	23.0 ± 3.9abC	39.4 ± 6.0bcB	76.3 ± 7.9aA	86.2 ± 7.2aA	46.9 ± 4.3bcB	Soil profile (S)
60–80	18.5 ± 4.0abB	59.3 ± 8.6bA	59.1 ± 9.4abA	74.1 ± 4.5abA	78.7 ± 12.4aA	*p* < 0.001
80–100	26.9 ± 3.8aC	96.5 ± 9.8aA	33.4 ± 5.3cC	57.8 ± 5.7bcB	69.7 ± 7.1aB	T × S
100–120	23.4 ± 5.1abC	50.8 ± 8.5bAB	35.6 ± 5.1cBC	64.1 ± 7.8bcA	26.4 ± 6.5cC	*p* < 0.001

Data are mean values ± standard error (SE). Different small letters within the same column and different capital letters within the same row for each treatment indicate a significant difference at *p* < 0.05, determined by Tukey’s multiple range tests.

**Table 3:** Cumulative leaching amount of nitrogen (NH_4_^+^-N, NO_3_^−^-N, total nitrogen [TN]) from different treatments in the summer maize growing season. CK: no fertilizer, CF: chemical fertilizer, HH: blackwater, CH1: combined application of chemical fertilizer and blackwater at ration of 1:1, CH2: combined application of chemical fertilizer and blackwater at ratio of 2:1.

Nitrogen Form	Cumulative Leaching Amount (kg·ha^−1^)				
	CK	CF	HH	CH1	CH2
NH_4_^+^-N	0.75 ± 0.12b	0.76 ± 0.13b	2.82 ± 0.73a	1.31 ± 0.53b	1.08 ± 0.32b
NO_3_^−^-N	21.61 ± 5.88b	40.62 ± 7.87a	37.63 ± 2.53a	36.52 ± 6.47a	34.10 ± 5.64a
TN	34.91 ± 4.81b	60.95 ± 11.00a	56.31 ± 16.47a	56.21 ± 7.57a	53.14 ± 10.50a

Data are mean values ± SE. Different small letters within the same row for each treatment indicate a significant difference at *p* < 0.05, determined by Tukey’s multiple range tests.
